# Low BOK Expression Promotes Epithelial–Mesenchymal Transition and Migration via the Wnt Signaling Pathway in Breast Cancer Cells

**DOI:** 10.3390/ijms26157252

**Published:** 2025-07-27

**Authors:** Ling Liu, Tiantian He, Zhen Zhang, Wenjie Dai, Liyang Ding, Hong Yang, Bo Xu, Yitong Shang, Yu Deng, Xufeng Fu, Xing Du

**Affiliations:** Key Laboratory of Fertility Preservation and Maintenance of Ministry of Education, School of Basic Medical Sciences, Ningxia Medical University, Yinchuan 750004, China

**Keywords:** breast cancer, epithelial–mesenchymal transition, migration, Bcl-2-related ovarian killer, Wnt

## Abstract

The B-cell lymphoma 2 (Bcl-2)-related ovarian killer (BOK), a member of the Bcl-2 protein family, shares a similar domain structure and amino acid sequence homology with the pro-apoptotic family members BAX and BAK. Although BOK is involved in the development of various types of cancer, its mechanism of action in breast cancer remains unclear. This study found that BOK was involved in the process of MG132, inhibiting the migration and epithelial–mesenchymal transition (EMT) of breast cancer cells induced by transforming growth factor-β. Furthermore, interfering BOK reversed the inhibition of breast cancer cell migration and the EMT process by MG132. Additional studies revealed that BOK silencing promoted the expression of EMT-related markers in breast cancer cells, while BOK overexpression inhibited EMT and migration. Using RNA-seq sequencing and Western blotting, we confirmed that the Wnt signaling pathway is involved in BOK regulating the EMT process in breast cancer cells. Therefore, we conclude that low BOK expression promotes breast cancer EMT and migration by activating the Wnt signaling pathway. This study enhances our understanding of breast cancer pathogenesis and suggests that BOK may serve as a potential prognostic marker and therapeutic target for breast cancer.

## 1. Introduction

Breast cancer is the most common malignancy and the leading cause of cancer-related deaths among women worldwide [1]. Although current diagnostic and treatment strategies can significantly improve the survival rates of patients with breast cancer, the effectiveness of inhibiting breast cancer metastasis and development is not optimistic [2]. Hence, an urgent need exists for novel and efficacious therapeutic strategies targeting breast cancer metastasis. Breast cancer initially manifests as a localized disease but frequently metastasizes to various vital organs, thereby emerging as the primary cause of breast cancer-related mortality [3]. The epithelial–mesenchymal transition (EMT) of cancer cells has been demonstrated to be the principal mechanism underlying the invasiveness and metastasis of various cancers. During the EMT process, epithelial cells lose the adhesion feature and acquire migratory and invasive properties; this transition is essential for numerous developmental processes and for initiating cancer metastasis [4]. Recent studies have confirmed that epithelial cadherin (E-cadherin) and vimentin, as pivotal molecular markers in the EMT process, are considered to be markers of the invasion of cancer cells [5,6]. The targeted regulation of EMT is considered a promising strategy for effectively inhibiting the invasion and migration of cancer cells.

Our previous studies demonstrated that the B-cell lymphoma 2 (Bcl2) gene encodes an anti-apoptotic protein crucial for regulating cell cycle arrest [7]. Moreover, increasing studies have indicated that member proteins of the Bcl-2 family are potential and promising targets for the treatment of cancer development [8]. The Bcl-2-related ovarian killer (BOK) protein is a member of the Bcl-2 family that regulates apoptosis. It shares a similar domain structure and amino acid sequence homology with the pro-apoptotic Bcl-2 family members BAX and BAK. The homeostasis of these anti-apoptotic and pro-apoptotic proteins in the Bcl-2 family collectively maintained cell survival and death [9]. The BOK plays a nonapoptotic role in regulating proliferation and exhibits protective roles in response to specific stressors [10,11,12]. However, numerous previous studies have highlighted that BOK has pro-apoptotic properties [13,14,15]. Although the role of BOK remains controversial, the mechanisms by which it regulates cancer cell invasion and migration are unclear. Additionally, a previous study provided evidence for the potential role of BOK as a tumor suppressor in cancer and considered that the BOK gene was silenced in many human cancers [16]. Studies have demonstrated that BOK is downregulated in early non-small cell lung cancer and colorectal cancer and has potential prognostic value [17,18]. Therefore, understanding the role of BOK in cancer is crucial.

This study first explored BOK expression in normal populations and patients with breast cancer and the correlation between the BOK expression level and overall survival based on the database. It then assessed breast cancer cell migration and EMT occurrence following BOK upregulation or downregulation. Finally, the underlying mechanism was identified and verified through transcriptomic analysis. Therefore, we propose the hypothesis that BOK plays a role in inhibiting metastasis in breast cancer. Its downregulated expression promotes the migration ability of breast cancer cells by regulating the epithelial–mesenchymal transition (EMT) process, ultimately leading to a poor prognosis for patients. This provides new insights into the role of BOK in developing breast cancer.

## 2. Results

### 2.1. Bioinformatics Analysis of BOK Expression and Prognostic Significance in Breast Cancer Using Public Databases

**Figure 1:**
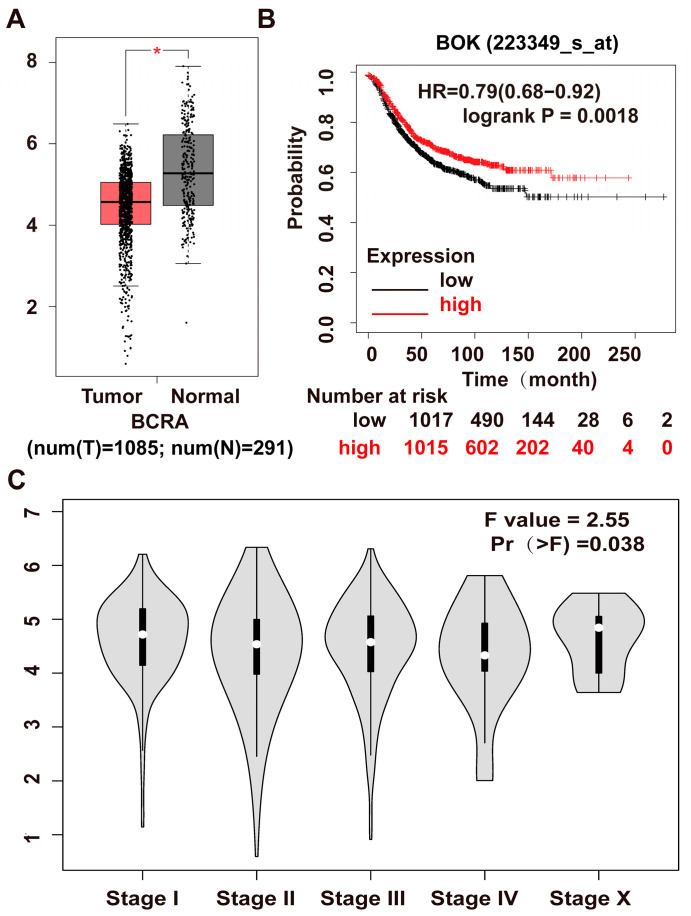
Examination of BOK expression in breast cancer and clinical prognosis using database analysis. (**A**) BOK expression in breast cancer tissues and paracancerous normal tissue from the GEPIA public database. (**B**) The relationship between BOK expression and survival rate of patients with breast cancer based on Kaplan–Meier plotter analysis. Red represents BOK high expression, and black represents BOK low expression. (**C**) BOK expression in various clinical stages of breast cancer from the GEPIA public database. Pr (>F) represents the *p*-value of the Fisher test. * *p* < 0.05.

To clarify BOK expression in breast cancer, the GEPIA (Gene Expression Profiling Interactive Analysis) public database was employed to analyze BOK expression in 1085 breast cancer tissues and 291 normal breast tissues via bioinformatics. The results revealed that BOK was significantly downregulated in 1085 cases of breast cancer tissues compared to 291 normal tissues (Figure 1A). In addition, the BOK expression level was not significantly different across different stages of breast cancer (Figure 1C). To investigate the prognostic significance of BOK expression in breast cancer, the relationship between BOK expression and the survival of patients with breast cancer was determined using the Kaplan–Meier Plotter (http://kmplot.com/analysis/index.php?p=service&cancer=breast_rnaseq_gse96058 (accessed on 12 January 2024) database (GSE96058). The results revealed that patients with high BOK expression exhibited prolonged survival time and increased survival probability (Figure 1B). These findings suggest that BOK is expressed at low levels in breast cancer tissues and is correlated with a poor prognosis, indicating its potential role as an anti-cancer gene in breast cancer.

### 2.2. BOK Is Involved in the Process of TGF-β-Induced EMT

**Figure 2:**
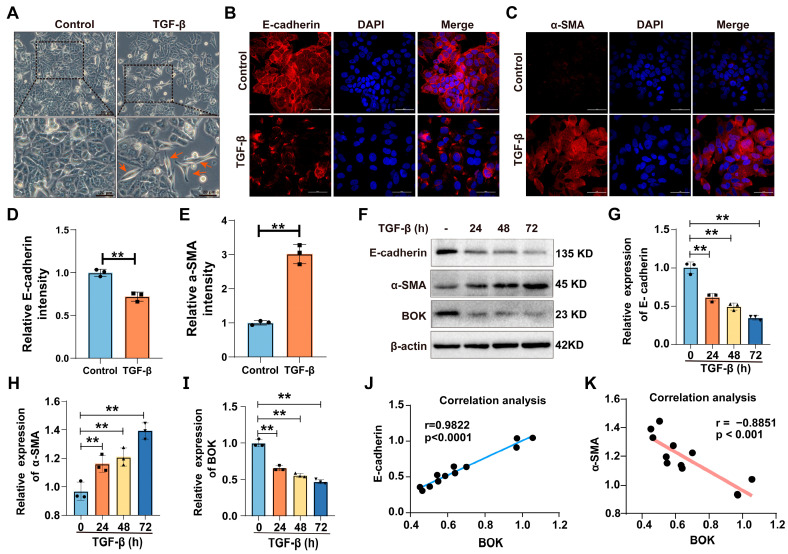
BOK participates in TGF-β-induced EMT process. (**A**) Morphology of TGF-β induction in MCF-7 cells. The control cells were arranged in a typical paving stone-like pattern. The cells treated with TGF-β were spindle-shaped (indicated by the red arrow) and had the characteristics of EMT; scale bar: 50 μm and 20 μm. (**B**,**C**) Immunofluorescence images of epithelial marker E-cadherin and mesenchymal marker α-SMA expression in MCF-7 cells induced by TGF-β for 72 h; scale bar: 50 μm. (**D**,**E**) Quantification of E-cadherin and α-SMA expression in (**B**,**C**). (**F**) Western blotting images of E-cadherin, α-SMA, and BOK expression in MCF-7 cells after TGF-β (10 ng/mL) treatment for 0, 24, 48, and 72 h. Quantification of E-cadherin (**G**), BOK (**H**), and α-SMA (**I**) expression in (**F**). Correlation between E-cadherin (**J**) and α-SMA (**K**) expression and BOK expression. ** *p* < 0.01.

Numerous studies have demonstrated the importance of the TGF-β signaling pathway in the initiation and metastasis of EMT in tumor cells [19,20,21]. Therefore, TGF-β (10 ng/mL) was used to induce the EMT model in MCF-7 cells. In morphology, MCF-7 cells in the control group were uniformly sized and arranged in the shape of a paving stone. However, the morphology of cells treated with TGF-β exhibited a transition to a spindle-shaped appearance, suggesting that epithelial–mesenchymal transition (EMT) had been induced in MCF-7 cells (Figure 2A). Furthermore, through immunofluorescence staining for E-cadherin and α-SMA expression, the results revealed that compared with the control group, the expression of E-cadherin was downregulated in the TGF-β treatment group (Figure 2B,D), while the expression of α-SMA was upregulated (Figure 2C,E). These results indicate that the TGF-β-induced EMT model has been successfully established.

To explore whether BOK is involved in the EMT process of MCF-7 cells, the expression of E-cadherin, α-SMA, and BOK was detected using Western blotting after TGF-β treatment for 0, 24, 48, and 72 h. The results revealed a time-dependent decrease in both E-cadherin and BOK expression levels after TGF-β treatment compared to the control group (Figure 2F,G,I). Conversely, α-SMA expression levels exhibited a time-dependent increase (Figure 2F,H). The results indicated that BOK expression is suppressed with an enhancement in the EMT process. Furthermore, by quantifying protein expression and analyzing the correlation between E-cadherin and α-SMA expression and BOK expression, the results demonstrated that E-cadherin was positively correlated with BOK expression, whereas α-SMA was negatively correlated with BOK expression (Figure 2J,K), indicating that BOK is involved in the process of TGF-β-induced EMT.

### 2.3. BOK Undergoes Ubiquitination Through the Proteasome Pathway

**Figure 3:**
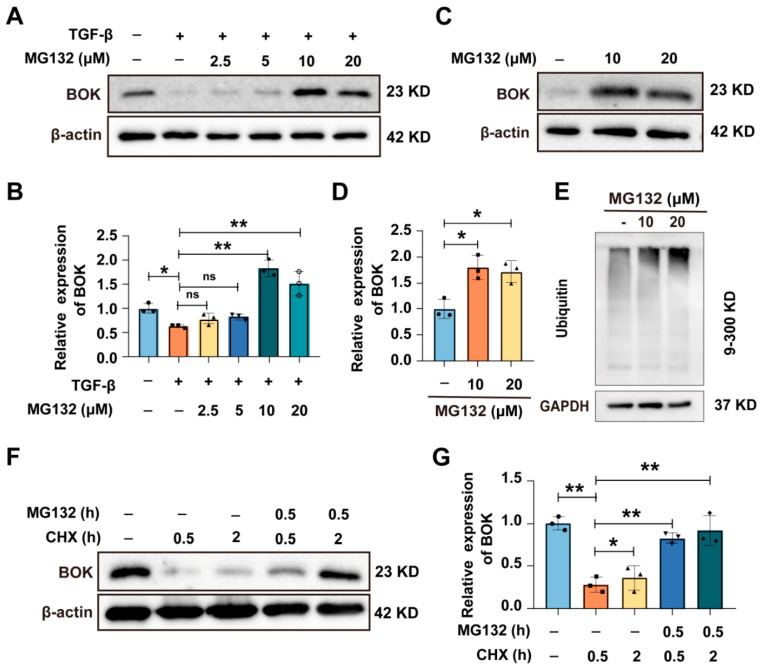
BOK undergoes ubiquitination through the proteasome pathway. (**A**) Expression of BOK in MCF-7 cells after treatment with TGF-β and MG132. (**B**) Quantification of BOK expression in (**A**). (**C**) Expression of BOK after MG132 (10 and 20 μM) treatment in MCF-7 cells. (**D**) Quantification of BOK expression in (**B**). (**E**) Overall ubiquitination levels after MG132 treatment in MCF-7 cells. (**F**) Expression of BOK after MG132 and CHX treatment for 0.5 and 2 h in MCF-7 cells. (**G**) Quantification of BOK expression in (**F**). *n* = 3, * *p* < 0.05, ** *p* < 0.01.

Studies have demonstrated that BOK is a constitutive pro-apoptotic factor, typically degraded via the ubiquitin–proteasome pathway (UPP) and maintained at low levels. Only when the UPP-dependent process is blocked will BOK accumulate, leading to cell apoptosis [22,23]. To further determine that BOK functions through ubiquitination in breast cancer, BOK expression was detected after a series of concentrations of proteasome inhibitor MG132 (2.5, 5, 10, and 20 μM) and TGF-β treatment in MCF-7 cells. The results demonstrated that the expression of BOK in MCF-7 cells was downregulated following treatment with TGF-β alone. However, when cells were co-treated with TGF-β and MG132, BOK expression was upregulated (Figure 3A,B). Furthermore, treatment with MG132 alone also led to increased BOK expression (Figure 3C,D). Meanwhile, the overall level of ubiquitination of proteins increased (Figure 3E). To further confirm that BOK is degraded through ubiquitination and depends on the proteasome pathway, BOK expression was detected after MG132 and cyclohexane (CHX) treatment in MCF-7 cells at 0.5 and 2 h. The results indicated that CHX inhibited the synthesis of BOK protein, while MG132 reversed the decrease in BOK caused by CHX (Figure 3F,G). These results suggest that BOK is degraded through ubiquitination via the proteasome pathway.

### 2.4. MG132 Inhibits the Process of Breast Cancer Cell Migration and EMT Induced by TGF-β

**Figure 4:**
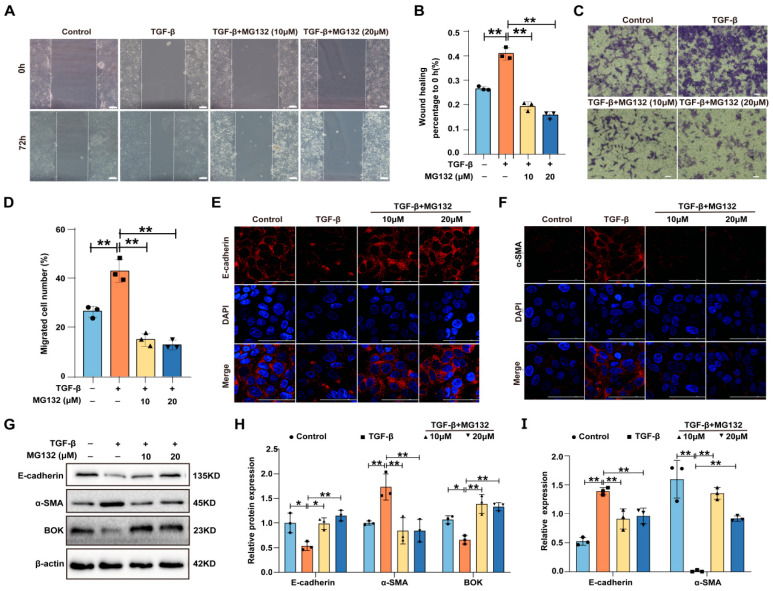
MG132 inhibits breast cancer cell migration and EMT induced by TGF-β. (**A**) Effects of MG132 and TGF-β on the wound healing ability of MCF-7 cells; scale bar: 100 μm. (**B**) Quantification of (**A**). (**C**) Migratory effects of MG132 and TGF-β treatment on MCF-7 cells evaluated using a Transwell migration assay; scale bar: 50 μm. (**D**) Quantification of (C). (**E**,**F**) Immunofluorescence images of E-cadherin (**E**) and α-SMA (**F**) expression in MCF-7 cells treated with MG132 and TGF-β; scale bar: 100 μm. (**G**) Western blotting images of E-cadherin, α-SMA, and BOK expression in MCF-7 cells treated with MG132 and TGF-β. (**H**) Quantification of (**G**). (**I**) Effects of MG132 and TGF-β on the mRNA levels of E-cadherin and α-SMA in MCF-7 cells. *n* = 3, * *p* < 0.05, ** *p* < 0.01.

Studies have revealed that proteasome inhibitor MG132 exhibits a significant effect in treating malignant tumors [24,25,26]. Wound healing and Transwell migration assays were used to evaluate MG132’s impact on TGF-β-induced migration in MCF-7 cells. The wound healing assay results demonstrated that MG132 could reduce the wound healing rate induced by TGF-β in MCF-7 cells (Figure 4A,B). The Transwell migration assay results revealed that MG132 could reduce the number of MCF-7 cell migrations induced by TGF-β (Figure 4C,D). Furthermore, the expression of EMT-related proteins (E-cadherin and α-SMA) was detected using immunofluorescence, and the results indicated that MG132 reversed the downregulation of E-cadherin and the upregulation of α-SMA induced by TGF-β in MCF-7 cells (Figure 4E,F). Subsequently, the expression of E-cadherin, α-SMA, and BOK was detected using Western blotting, with the results demonstrating that the expression of E-cadherin and α-SMA was reversed by MG132 in TGF-β-induced MCF-7 cells (Figure 4G,H). Meanwhile, the mRNA expression levels were detected by RT-qPCR, and the trends paralleled the protein expression patterns (Figure 4I). These results suggest that BOK is crucial in inhibiting the migration and EMT processes of breast cancer cells induced by TGF-β under MG132 treatment.

### 2.5. Knockdown of BOK Promotes EMT and Migration Ability in MCF-7 and MDA-MB-231 Cells

**Figure 5:**
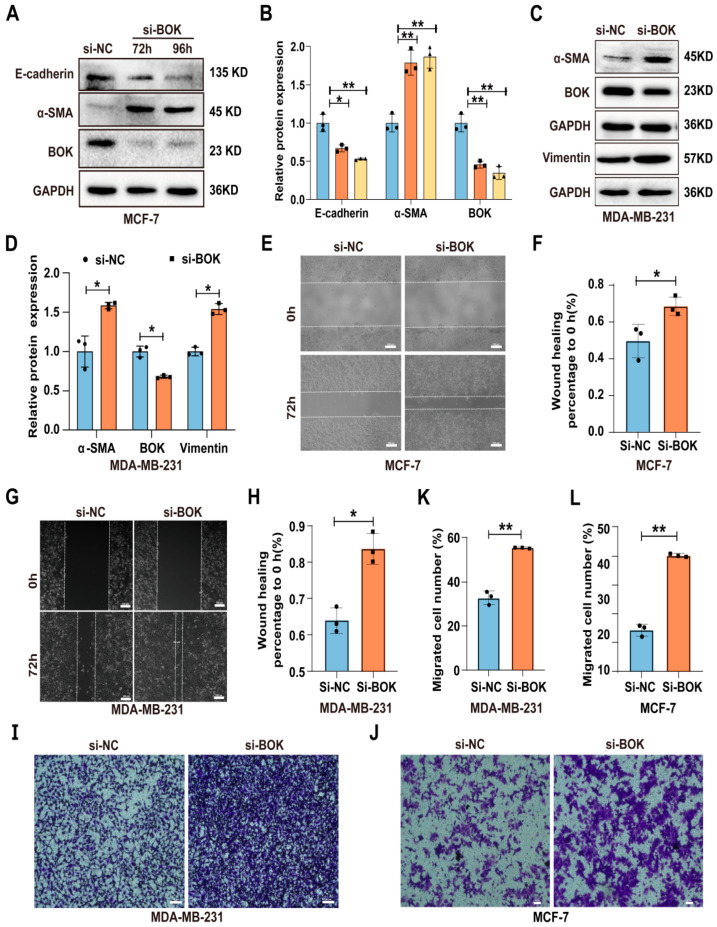
Knockdown of BOK promotes EMT and migration ability in MCF-7 and MDA-MB-231 cells. (**A**–**D**) Expression and quantification of EMT-related proteins after BOK knockdown in MCF-7 and MDA-MB-231 cells. (**E**–**H**) Wound healing images and quantification after BOK knockdown in MCF-7 and MDA-MB-231 cells; scale bar: 100 μm. (**I**–**L**) Transwell images and quantification after BOK knockdown in MCF-7 (scale bar: 50 μm) and MDA-MB-231 cells (scale bar: 100 μm). *n* = 3, * *p* < 0.05, ** *p* < 0.01.

To further investigate BOK’s role in breast cancer cells, BOK was knocked down in MCF-7 and MDA-MB-231 cells and detected using Western blotting and RT-qPCR. The results indicated that BOK expression was successfully knocked down in both MCF-7 and MDA-MB-231 cells at the translation (Figure S1A–D) and transcription (Figure S1E,F) levels. Furthermore, the expression of EMT-related proteins in MCF-7 and MDA-MB-231 cells after BOK knockdown was detected using Western blotting. The results revealed that E-cadherin expression in the si-BOK group was lower, whereas α-SMA expression was higher than that in the control group in MCF-7 cells (Figure 5A,B). In MDA-MB-231 cells, α-SMA and vimentin expression were significantly upregulated in the si-BOK group compared with the control group (Figure 5C,D). These results suggested that BOK knockdown can enhance the EMT process in MCF-7 and MDA-MB-231 cells. Subsequently, the wound healing assay results suggested that the wound healing ability of si-BOK in MCF-7 and MDA-MB-231 cells was significantly stronger than that of the control group (Figure 5E–H). Additionally, the Transwell migration assay results indicated that the number of MCF-7 and MDA-MB-231 cells that passed through the Transwell chambers in the si-BOK group was significantly higher than that in the control group (Figure 5I–L). These results indicated that BOK knockdown can enhance the EMT and migration ability in MCF-7 and MDA-MB-231 cells.

### 2.6. BOK Knockdown Can Reverse the Inhibition of MG132 on the Migration and the EMT Process in MCF-7 Cells

**Figure 6:**
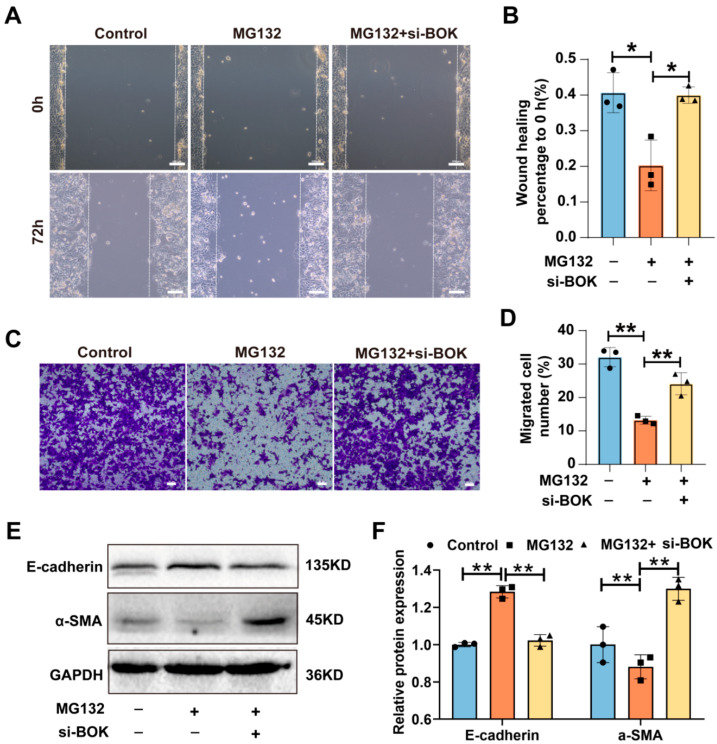
BOK knockdown reverses MG132 inhibition of migration and the EMT process in MCF-7 cells. (**A**) Images of the wound healing assay with BOK knockdown following MG132 treatment in MCF-7 cells; scale bar: 100 μm. (**B**) Quantification of (**A**). (**C**) Images of the Transwell assay with BOK knockdown following MG132 treatment in MCF-7 cells; scale bar: 50 μm. (**D**) Quantification of (**C**). (**E**) E-cadherin and α-SMA expression with BOK knockdown following MG132 treatment in MCF-7 cells. (**F**) Quantification of (**E**). *n* = 3, * *p* < 0.05, ** *p* < 0.01.

To further elucidate the BOK-mediated antitumor effects of proteasome inhibitors, MCF-7 cells were treated with MG132 and knockdown BOK to investigate the migration ability and EMT process. The wound healing assay results suggested that MG132 reduced the wound healing rate in MCF-7 cells compared to the control, while BOK knockdown reversed the inhibitory effect of MCF-7 cell migration caused by MG132 (Figure 6A,B). Likewise, the Transwell migration assay results demonstrated that MG132 reduced the number of cell migrations compared to the control, and BOK knockdown rescued this effect (Figure 6C,D). Subsequently, EMT-related protein expression was detected using Western blotting, and the results revealed that E-cadherin expression was upregulated and α-SMA was downregulated after MG132 treatment in breast cancer MCF-7 cells compared to the control. However, after BOK knockdown, α-SMA expression was upregulated, while E-cadherin expression was downregulated (Figure 6E,F). These findings suggest that BOK knockdown can reverse the inhibitory effects of MG132 on MCF-7 cell migration and the EMT process, providing further evidence of BOK involvement in the antitumor activity of MG132.

### 2.7. BOK Overexpression Inhibits the EMT Process and Migration Ability in MCF-7 and MDA-MB-231 Cells

**Figure 7:**
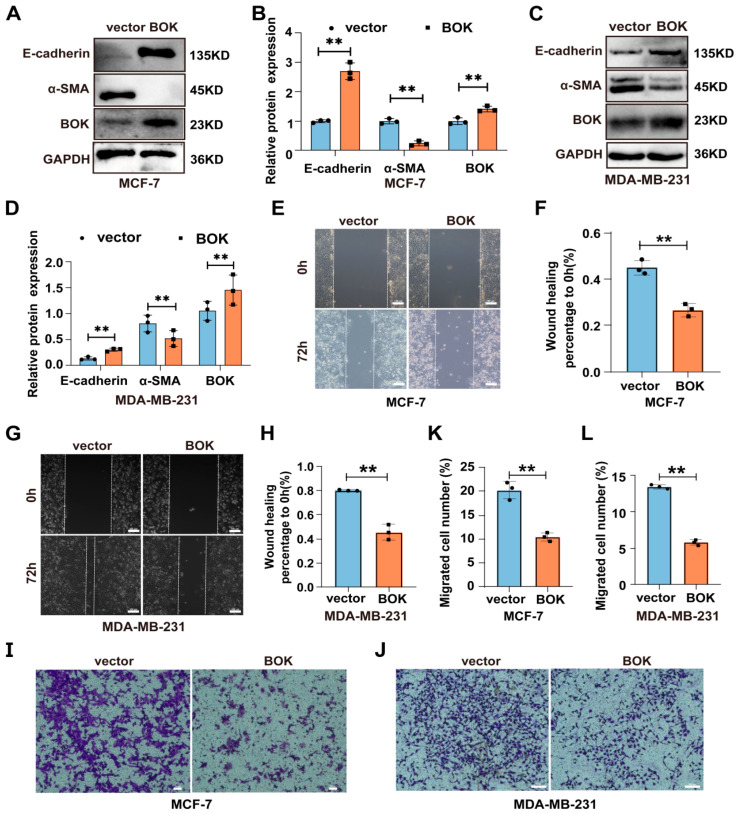
BOK overexpression inhibits the EMT process and migration ability in MCF-7 and MDA-MB-231 cells. (**A**–**D**) Expression and quantification of EMT-related proteins after BOK overexpression in MCF-7 and MDA-MB-231 cells. (**E**–**H**) Wound healing images and quantification after BOK overexpression in MCF-7 and MDA-MB-231 cells; scale bar: 100 μm. (**I**–**L**) Transwell images and quantification after BOK overexpression in MCF-7 (scale bar: 50 μm) and MDA-MB-231 cells (scale bar: 100 μm). *n* = 3, ** *p* < 0.01.

To further investigate BOK’s role in regulating the EMT process and migration of breast cancer cells, MCF-7 and MDA-MB-231 cells were transfected with an empty vector (pcDNA) and overexpression vector (pcDNA-BOK), respectively. Additionally, the successful construction of BOK overexpression cell lines in MCF-7 and MDA-MB-231 cells was verified using Western blotting and RT-qPCR methods (Figure S2), which could be used for subsequent detection. Furthermore, the overexpression of EMT-related proteins in BOK-overexpressing MCF-7 and MDA-MB-231 cells was detected using Western blotting. The result suggested that E-cadherin is upregulated and α-SMA is downregulated after BOK overexpression in MCF-7 (Figure 7A,B) and MDA-MB-231 (Figure 7C,D) cells. This indicated that BOK overexpression inhibits the EMT process in MCF-7 and MDA-MB-231 cells. Subsequently, the migration of two breast cancer cells was detected using wound healing and Transwell migration assays after BOK overexpression. The results revealed that the wound healing ability of the BOK overexpression group in MCF-7 and MDA-MB-231 cells was weaker than that of the control group (Figure 7E–H). Additionally, the number of MCF-7 and MDA-MB-231 cells that passed through the Transwell chambers in the BOK overexpression group was significantly lower than that in the control group (Figure 7I–L). These results suggest that BOK overexpression inhibits EMT and the migration of breast cancer cells.

### 2.8. Wnt/β-Catenin Signaling Pathway Activation May Represent a Crucial Mechanism by Which BOK Regulates the Migration and EMT in MCF-7 Cells

**Figure 8:**
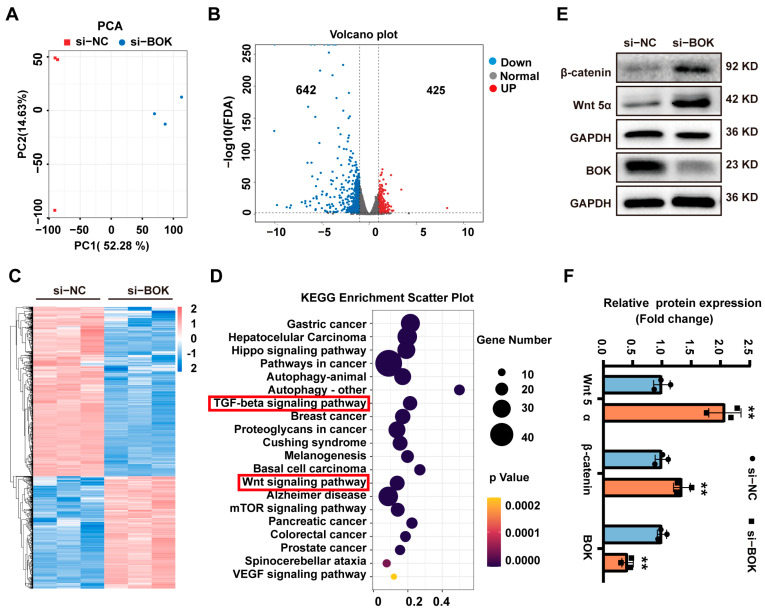
BOK regulates the migration and EMT of MCF-7 cells through the Wnt/β-catenin signaling pathway. (**A**) PCA evaluation plot and (**B**) volcano plot for control and si-BOK groups, with red dots representing upregulated genes and blue dots representing downregulated genes. (**C**) Heatmap of differentially expressed genes, with red indicating upregulated genes and blue representing downregulated genes. (**D**) Bubble plot of KEGG pathway enrichment. (**E**) Western blotting image of Wnt5α, β-catenin, and BOK expressions. (**F**) Quantification of (**E**). *n* = 3, ** *p* < 0.01.

To investigate the mechanism by which BOK regulates breast cancer cell migration and EMT, gene expression in MCF-7 cells after BOK knockdown was analyzed using RNA-seq sequencing. The principal component analysis (PCA) evaluation results suggested that the gene expression levels in the si-BOK and control groups differed significantly (Figure 8A). Additionally, the volcano plot indicated 1067 differentially expressed genes in the si-BOK group compared to the transfected empty vector group (control), with 642 genes downregulated and 425 genes upregulated (Figure 8B). These differential genes were exhibited in a heatmap (Figure 8C). Furthermore, the differentially expressed genes were enriched in the TGF-β and Wnt pathways through KEGG enrichment analysis (Figure 8D). Interestingly, our experimental results revealed that BOK expression was downregulated in a time-dependent manner with the prolonged TGF-β treatment (Figure 2F,I), which paralleled the results of the KEGG enrichment analysis. In addition, these results suggested that the Wnt signaling pathway may be a key mechanism by which low BOK expression leads to MCF-7 cell migration and EMT. In addition, this result confirmed that BOK is involved in MCF-7 cell migration and the EMT process. To further verify the sequencing results of RNA-seq, the expression of Wnt5α and β-catenin in the Wnt signaling pathway was further detected using Western blotting after BOK knockdown. The result indicated that the expression of Wnt5α and β-catenin in MCF-7 cells after BOK knockdown was upregulated (Figure 8E,F). These results suggest that low expression of BOK may regulate migration and EMT by activating the Wnt/β-catenin signaling pathway in MCF-7 cells.

## 3. Discussion

This study demonstrated that BOK suppresses the migration of breast cancer cells by inhibiting EMT. Moreover, using the GEPIA and Kaplan–Meier plotter database analyses, we found that BOK is lowly expressed in patients with breast cancer, and patients with high BOK expression exhibit longer survival times and increased survival probabilities. This suggests that BOK is a promising prognostic marker and therapeutic target for breast cancer. The BCL-2 family proteins, comprising pro- and anti-apoptotic members, are crucial in targeted therapies for cancer progression [27,28]. As BOK is a member of the BCL-2 family and homologous to pro-apoptotic BAX and BAK sequences, its role is being studied in human tumors [17,18]. In a previous somatic copy-number alteration investigation involving 3131 cancer specimens, BOK was absent in numerous types of human cancer, indicating that BOK may exert a tumor-suppressing role in human cancers [16]. Numerous studies have demonstrated BOK involvement in the pathogenesis of various cancer types, indicating its potential anti-cancer effects in breast [29], liver [30], non-small cell lung [18], and colorectal cancers [17]. In addition, BOK expression has been observed to be deficient in several multiple myeloma cell lines [31]. As demonstrated by our study, BOK plays an inhibitory role in breast cancer progression. However, the role of BOK in regulating tumor initiation and progression remains unclear. Increasing studies have shown that TGF-β promotes the EMT process of cancer cells [32,33].

As TGF-β is crucial in the induction of EMT in tumor cells, cell models utilizing TGF to induce EMT have been widely employed [33,34]. Consequently, to further investigate BOK’s role in the EMT process of breast cancer cells, this study employed the TGF-β-induced EMT process in MCF-7 cells, and the result suggested that BOK expression was downregulated in a time-dependent manner with the prolonged TGF-β treatment. Similarly, in a study of non-small cell lung carcinoma, BOK inhibited the EMT process, served as a tumor suppressor, and attenuated the capacity of TGF-β to induce cellular migration [18]. Furthermore, BOK knockdown in pancreatic cancer cells enhanced migration and invasion ability [35]. As a result, BOK may play a key role in regulating the EMT process and impacting the progression of breast cancer cells. Additionally, endogenous BOK is consistently detectable across all cell types, including mouse embryonic fibroblasts (MEFs), and its expression level remains unchanged following treatment with proteasome inhibitors.

Endogenous BOK is relatively stable on the endoplasmic reticulum membrane, and its expression does not change with the addition of proteasome inhibitors [36]. BOK, as a widely expressed apoptosis regulatory factor, maintains a basal expression level in mammalian cells [37,38]. In this present study, BOK underwent ubiquitin-mediated degradation in breast cancer cells, and MG132 has been shown to inhibit the EMT process and the migration of MCF-7 cells. However, BOK knockdown reverses this effect, thereby confirming that the ubiquitin-mediated degradation of BOK plays a role in regulating the EMT process and migratory capacity of breast cancer cells. In addition, this study employed scratch wound healing assays, Transwell assays, and the analysis of EMT markers (including E-cadherin, α-SMA, and vimentin) to assess the effects of BOK silencing and overexpression in both MCF-7 and MDA-MB-231 cell lines. The experimental results further support that BOK suppresses the EMT process in breast cancer cells. Similar to this study, studies have demonstrated that UPP typically regulates BOK expression to maintain low levels [22,23]. Moreover, the proteasome inhibitor MG132 has a significant therapeutic effect against malignant tumors [24,25,26]. This study demonstrated that BOK undergoes ubiquitination-mediated degradation in breast cancer cells and that MG132 inhibits the EMT process and migration ability in MCF-7 cells. However, BOK knockdown can reverse these phenomena, confirming that BOK ubiquitination-mediated degradation is involved in the EMT process and migration of breast cancer cells. Furthermore, BOK was silenced and overexpressed in MCF-7 and MDA-MB-231 cell lines, respectively, using the wound healing assay, Transwell assay, and detection of EMT markers (E-cadherin, α-SMA, and vimentin). These results confirm that BOK inhibits the EMT process in breast cancer cells. Similarly, miR-296-5p targets BOK to participate in invasion and EMT in pancreatic cancer [35]. Notably, BOK functions as a tumor suppressor in non-small cell lung carcinoma by inhibiting EMT [18]. Therefore, the ubiquitination of BOK may play a role in the EMT process and the migration of breast cancer cells.

To further investigate the mechanism by which low BOK expression promotes the EMT and migration ability of breast cancer cells, we found that the differentially expressed genes were significantly enriched in the Wnt signaling pathway. Using Western blotting, we further confirmed that the expression of Wnt5α and β-catenin was upregulated in MCF-7 cells with low BOK expression. The Wnt signaling pathway is essential in the proliferation and metastasis of breast cancer [39]. Consistent with this, the sustained activation of the Wnt pathway is associated with treatment resistance in cancer patients and has been shown to promote the self-renewal of cancer cells [40]. A study of whole-exome sequencing of duodenal adenocarcinoma found recurrent mutations in BOK that involve the Wnt/β-catenin pathway [41]. Additionally, disruption of WNT signaling significantly decreased BOK expression in the colorectal cancer cell line Ls174T, indicating that Wnt signaling controls the expression of pro-apoptotic BOK [42]. However, the detailed mechanism by which BOK regulates the Wnt signaling pathway during the EMT process in breast cancer cells still needs further investigation.

In conclusion, low expression of BOK may contribute to the activation of the Wnt signaling pathway, which in turn mediates the epithelial–mesenchymal transition (EMT) process in breast cancer cells and enhances their migratory capacity. Furthermore, this study contributed to an enhanced comprehension of the mechanisms underlying breast cancer metastasis and proposed BOK as a promising prognostic marker and therapeutic target for breast cancer.

## 4. Materials and Methods

### 4.1. Cell Culture and Transfection

Human breast cancer cell lines Michigan Cancer Foundation-7 (MCF-7) and MD Anderson–metastatic breast-231 (MDA-MB-231) were obtained from the Shanghai Cell Bank of the Chinese Academy of Sciences. In transfection, 6 µL of interfering ribonucleic acid (RNA) fragment (50 pM) and 6 µL of Lip2000 (Thermo Scientific, Waltham, MA, USA) were diluted in 200 µL serum-free medium for BOK knockdown experiments. The BOK overexpression plasmid and control vector pcDNA were obtained from Shanghai GenePharma Co., Ltd. (Shanghai, China). Furthermore, 2 µg DNA plasmid and 4 μL Lip2000 were diluted in 200 μL serum-free medium for overexpression transfection.

### 4.2. Total RNA Isolation and Reverse-Transcription Quantitative Polymerase Chain Reaction (RT-qPCR)

Total RNA was isolated from the cultured cells using TRIzol reagent (Invitrogen, Carlsbad, CA, USA). Subsequently, 1 µg of RNA was reverse transcribed into complementary DNA (cDNA) using an RT Kit (Takara Biotechnology, Shiga, Japan). Quantitative RT-PCR (RT-qPCR) was performed using gene-specific primers (Shengon, Shanghai, China) and TBGreen^®^PremeixEXTaqTMII (Takara, Shiga, Japan) on quantitative PCR analysis equipment (Bio-Rad, Richmond, CA, USA). The primers used in this study are listed in Table S1. Relative gene expression was calculated using the 2^−ΔΔCT^ method, with β-actin and GAPDH as internal controls.

### 4.3. Western Blotting Analysis

The MCF-7 or MB-MDA-231 cells were lysed to extract protein using a lysis buffer. Sodium dodecyl sulfate-polyacrylamide gel electrophoresis was employed to separate the proteins, then transferred onto a polyvinylidene fluoride membrane (Millipore, Burlington, MA, USA). The membrane was then blocked with 5% skim milk for 1 h and incubated with primary antibodies overnight at 4 °C. Afterward, the membranes were incubated with horseradish peroxidase-conjugated goat anti-rabbit or goat anti-mouse secondary antibodies (Thermo Fisher Scientific, Waltham, MA, USA) for 1 h at room temperature. The signal was detected with a fluorescence detection device (Thermo Scientific, USA). The grayscale values of the proteins were quantified using ImageJ software(version 1.54p). The relative intensity of the target proteins was normalized using β-actin and GAPDH. Table S2 displays the primary antibodies and their dilution ratios used in this experiment.

### 4.4. Wound Healing Assay

The MDA-MB-231 or MCF-7 cells were cultured for 24 h in a serum-free medium; then, a line was made through the cells to simulate an injury using a 2 mm wide pipette tip when cells reached 90% confluency. Subsequently, to remove detached cells, they were washed three times with phosphate-buffered saline and allowed to migrate in a serum-free medium. To measure cell migration, photographs were taken (at a magnification of 400×) after 72 h of MDA-MB-231 or MCF-7 cell growth. Three fields were chosen randomly for quantitative measurements within the injured areas.

### 4.5. Transwell Assay

MCF-7 or MDA-MB-231 cells were seeded in the upper chamber of a Transwell containing serum-free medium at a cell density of 5 × 10^4^ or 2 × 10^4^ cells. After 48 h of culturing, the chambers were stained with 0.5% gentian violet and photographed. Each experiment was repeated three times.

### 4.6. Immunofluorescence Analysis

After transfection with plasmid and treatment with transforming growth factor-β (TGF-β) (PeproTech, Rocky Hill, NJ, USA) and MG132 (MedChemExpress, Monmouth Junction, NJ, USA), MCF-7 cell crawling tablets were fixed for 15–20 min. Following blocking with goat serum for 1 h, the crawling tablets were incubated with E-cadherin (Cell Signaling Technology, Danvers, MA, USA) and α-smooth muscle actin (α-SMA; Affinity Biosciences, Melbourne, VIC, Australia) antibodies overnight at 4 °C. The cells were then incubated with the corresponding secondary antibody for 1 h at room temperature. Finally, the nuclei were stained with 4′, 6-diamidino-2-phenylindole (Beyotime, Shanghai, China) for 15 min and sealed. All images were observed and analyzed using a Nikon A1R microscope (Melville, NY, USA) with NIS-Elements Viewer 4.5 software.

### 4.7. Transcriptomic Analysis

Transcriptomic sequencing technology was used to sequence MCF-7 cell samples and detect BOK knockdown effects on difference-related gene expression in breast cancer MCF-7 cells. The samples were sent to Beijing Biomarker Technologies Co., Ltd. (Beijing, China) for subsequent steps and sequencing analysis.

### 4.8. Statistical Analysis

All statistical analyses were performed using GraphPad Prism (version 9.0). Before statistical analyses, *t*-tests were used to analyze differences between the two groups. For multiple comparisons, one-way or two-way analysis of variance combined with Dunnett’s or Tukey’s tests was used. Data are presented as the mean ± standard deviation of at least three independent experiments. For all analyses, *p* < 0.05 was considered statistically significant. The results were replicated in at least three independent trials.

## Data Availability

The data that support the findings of this study are available from the corresponding author upon reasonable request.

## Supplementary Materials

The following supporting information can be downloaded at: https://www.mdpi.com/article/10.3390/ijms26157252/s1.
