# Supplementary material for: Low BOK Expression Promotes Epithelial–Mesenchymal Transition and Migration via the Wnt Signaling Pathway in Breast Cancer Cells
Source: Int J Mol Sci. 2025 Jul 27;26(15):7252. doi: 10.3390/ijms26157252 (PMC12347190; doi:10.3390/ijms26157252)
Supplement: Supplementary file 1 [file ijms-26-07252-s001.zip › ijms-3724117-supplementary.pdf]

Supplemental imformation

Materials and methods

1. Cell culture and transfection

Human breast cancer cell lines Michigan Cancer Foundation-7 (MCF-7) and MD Anderson–metastatic breast-231 (MDA-MB-231) were obtained from the Shanghai Cell Bank of the Chinese Academy of Sciences. In transfection, 6 μL of interfering ribonucleic acid (RNA) fragment (50 pM) and 6 μL of Lip2000 (Thermo Scientific, USA) were diluted in 200 μL serum-free medium for BOK knockdown experiments. The BOK overexpression plasmid and control vector pcDNA were obtained from Shanghai GenePharma Co., Ltd. Furthermore, 2 μg DNA plasmid and 4 μL Lip2000 were diluted in 200 μL serum-free medium for overexpression transfection.

2. Total RNA isolation and reverse transcription–polymerase chain reaction (RT-PCR)

Total RNA was isolated from the cultured cells using TRIzol reagent (Invitrogen, USA). Subsequently, 1 μg of RNA was reverse transcribed into complementary DNA (cDNA) using an RT Kit (Takara Biotechnology, Japan). Quantitative RT-PCR (RT-qPCR) was performed using gene-specific primers (Shengon, China) and TBGreen®PremeixEXTaqTMII (Takara, Japan) on quantitative PCR analysis equipment (Bio-Rad, USA). The primers used in this study are listed in Table S1. Relative gene expression was calculated using the $2^{-\Delta\Delta CT}$ method, with β-actin and GAPDH as internal controls.

3. Western blotting analysis

The MCF-7 or MB-MDA-231 cells were lysed to extract protein using a lysis buffer . Sodium dodecyl sulfate–polyacrylamide gel electrophoresis was employed to separate the proteins, then transferred onto a polyvinylidene fluoride membrane (Millipore, USA). The membrane was then blocked with 5% skim milk for 1 h and

incubated with primary antibodies overnight at 4 °C. Afterward, the membranes were incubated with horseradish peroxidase-conjugated goat anti-rabbit or goat anti-mouse secondary antibodies (Thermo Fisher Scientific, USA) for 1 h at room temperature. The signal was detected with a fluorescence detection device (Thermo Scientific, USA). The grayscale values of the proteins were quantified using ImageJ software. The relative intensity of the target proteins was normalized using β-actin and GAPDH. Table S2 displays the primary antibodies and their dilution ratios used in this experiment.

4. Wound healing assay

The MDA-MB-231 or MCF-7 cells were cultured for 24 h in a serum-free medium; then, a line was made through the cells to simulate an injury using a 2 mm wide pipette tip when cells reached 90% confluency. Subsequently, to remove detached cells, they were washed three times with phosphate-buffered saline and allowed to migrate in a serum-free medium. To measure cell migration, photographs were taken (at a magnification of 400×) after 72 h of MDA-MB-231 or MCF-7 cell growth. Three fields were chosen randomly for quantitative measurements within the injured areas.

5. Transwell assay

MCF-7 or MDA-MB-231 cells were seeded in the upper chamber of a transwell containing serum-free medium at a cell density of 5×10^4 or 2×10^4 cells. After 48 h of culturing, the chambers were stained with 0.5% gentian violet and photographed. Each experiment was repeated three times.

6. Immunofluorescence analysis

After transfection with plasmid and treatment with transforming growth factor-β (TGF-β) (PeproTech, USA) and MG132 (MedChemExpress, USA), MCF-7 cell crawling tablets were fixed for 15–20 min. Following blocking with goat serum for 1 h, the crawling tablets were incubated with E-cadherin (Cell Signaling Technology, USA) and α-smooth muscle actin (α-SMA; Affinity Biosciences, Australia) antibodies

overnight at 4 °C. The cells were then incubated with the corresponding secondary antibody for 1 h at room temperature. Finally, the nuclei were stained with 4',6-diamidino-2-phenylindole (Beyotime, Shanghai, China) for 15 min and sealed. All images were observed and analyzed using a Nikon A1R microscope with NIS-Elements Viewer 4.5 software.

7. Transcriptomic analysis

Transcriptomic sequencing technology was used to sequence MCF-7 cell samples and detect BOK knockdown effects on difference-related gene expression in breast cancer MCF-7 cells. The samples were sent to Beijing Biomarker Technologies Co., LTD. for subsequent steps and sequencing analysis.

8. Statistical analysis

All statistical analyses were performed using GraphPad Prism (version 9.0). Before statistical analyses, t-tests were used to analyze differences between the two groups. For multiple comparisons, one-way or two-way analysis of variance combined with Dunnett's or Tukey's tests were used. Data are presented as the mean ± standard deviation of at least three independent experiments. For all analyses, $P < 0.05$ was considered statistically significant. The results were replicated in at least three independent trials.

Table S1. Sequences of oligonucleotide primers for QRT-PCR.

Gene Name	Forward Primer	Reverse Primer
β-actin	5'-AACCGCGAGAAGATGACCCAG-3'	5'-GGATAGCACAGCCTGGATAGCAA-3'
BOK	5'- AAGGTGGTGTCCCTGTATGC - 3'	5'- TGAGGACATCAGTCCATCCG - 3'
E-cadherin	5′-CTGGCGTCTGTAGGAAGGCA-3′	5′-GGGCAGTAAGGGCTCTTTGAC-3′
α-SMA	5'-GTGTTGCCCCTGAAGAGCAT-3'	5'-GCTGGGACATTGAAAGTCTCA-3'
GAPDH	5'-CAAATTCCATGGCACCGTCAA-3'	5'-AGCATCGCCCCACTTGATTT-3'

Table S2. Antibody informations in this study.

Antibody	Cat.No.	Company	Usage (Dilution)	Species
GAPDH	AF0006	Beyotime	WB (1:2000)	Mouse
β-actin	AF0003	Beyotime	WB (1:2000)	Mouse
E-cadherin	14472	CST	WB (1:1000), IF (1:200)	Rabbit
Vimentin	5741	CST	WB (1:1000)	Rabbit
α-SMA	AF1032	Affinity	WB (1:1000), IF (1:200)	Rabbit
BOK	ab233072	abcam	WB (1:1000)	Rabbit
Wnt5α	AF8358	Beyotime	WB (1:1000)	Rabbit
β-catenin	CY3523	Abways	WB (1:1000)	Rabbit
Ubiquitin	43124	CST	WB (1:1000)	Rabbit

WB: Western Blot. IF: immunofluorescence microscopy.

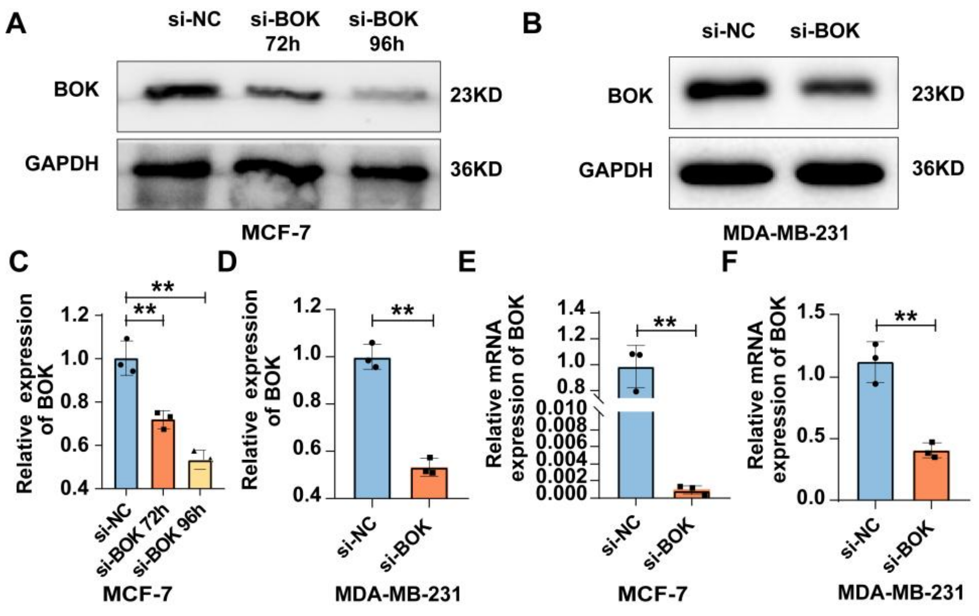

Figure S1 Identification of transient knockdown BOK expression in MCF-7 and MDA-MB-231 breast cancer cells. (A-D) Expression of BOK in MCF-7 cells and MDA-MB-231 after translation. (E-F)The expression level of BOK was detected by qRT-PCR after transient BOK knockdown. n=3, *P<0.05, **P<0.01.

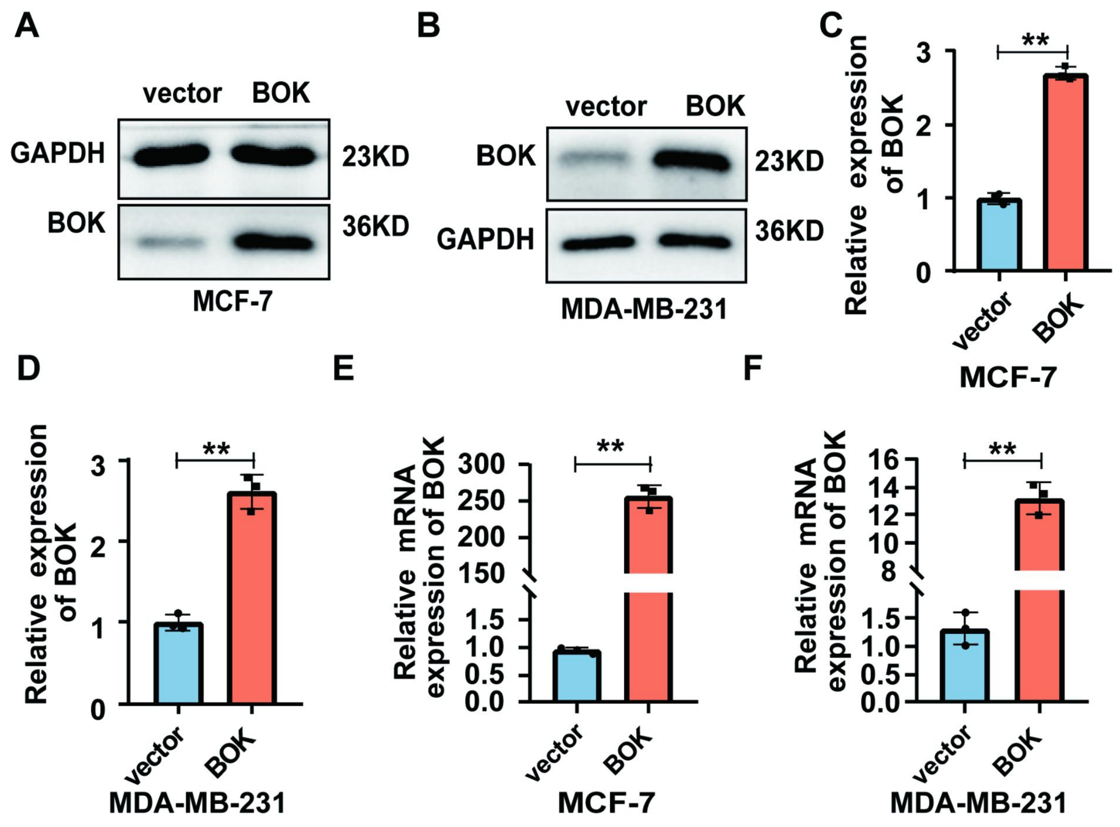

Figure S2 Identification of BOK overexpression in MCF-7 and MDA-MB-231 breast cancer cells. (A-D) Western Blot analysis of BOK expression level and its quantitative analysis

diagram after BOK overexpression; (E-F) The expression level of BOK was detected by qRT-PCR after BOK was overexpressed. n=3, *P<0.05, **P<0.01
